# Citrus Polyamines: Structure, Biosynthesis, and Physiological Functions

**DOI:** 10.3390/plants9040426

**Published:** 2020-03-31

**Authors:** Nabil Killiny, Yasser Nehela

**Affiliations:** 1 Citrus Research and Education Center and Department of Plant Pathology, IFAS, University of Florida, Lake Alfred, FL 33850, USA; yasser.nehela@ufl.edu; 2 Department of Agricultural Botany, Faculty of Agriculture, Tanta University, Tanta 31527, Egypt

**Keywords:** citrus, polyamines, putrescine, spermidine, spermine, embryogenesis, root system architecture, shoot system architecture, flowering and inflorescence

## Abstract

Polyamines (PAs) are ubiquitous biogenic amines found in all living organisms from bacteria to Archaea, and Eukaryotes including plants and animals. Since the first description of putrescine conjugate, feruloyl-putrescine (originally called subaphylline), from grapefruit leaves and juice, many research studies have highlighted the importance of PAs in growth, development, and other physiological processes in citrus plants. PAs appear to be involved in a wide range of physiological processes in citrus plants; however, their exact roles are not fully understood. Accordingly, in the present review, we discuss the biosynthesis of PAs in citrus plants, with an emphasis on the recent advances in identifying and characterizing PAs-biosynthetic genes and other upstream regulatory genes involved in transcriptional regulation of PAs metabolism. In addition, we will discuss the recent metabolic, genetic, and molecular evidence illustrating the roles of PAs metabolism in citrus physiology including somatic embryogenesis; root system formation, morphology, and architecture; plant growth and shoot system architecture; inflorescence, flowering, and flowering-associated events; fruit set, development, and quality; stomatal closure and gas-exchange; and chlorophyll fluorescence and photosynthesis. We believe that the molecular and biochemical understanding of PAs metabolism and their physiological roles in citrus plants will help citrus breeding programs to enhance tolerance to biotic and abiotic stresses and provide bases for further research into potential applications.

## 1. Introduction

Polyamines (PAs) are low molecular weight, small, linear/aliphatic and occasionally branched polycationic nitrogen compounds containing unsaturated hydrocarbon with two or more primary amino groups [1,2,3,4,5,6]. At physiological pH, the characteristic feature of PAs structure is that they are positively charged at a defined distance and have methylene groups harbored within them. These methylene groups are known to participate in hydrophobic interactions, thereby influencing PA activity as a whole [7]. In addition, due to the cationic nature of PAs, they can bind to negatively-charged macromolecules, such as nucleic acids (DNA and RNA), acidic phospholipids, and numerous types of proteins [3] to stabilize their structure, particularly under stress conditions.

The history of PAs is about a hundred and thirty years older than that of proteinogenic amino acids. Asparagine was the first isolated amino acid in 1806 [8], whereas, the first polyamine was discovered in 1678 and was subsequently named “spermine” [9]. In that year, Antonie van Leeuwenhoek described the presence of crystalline substances in human semen after standing for several days, but not observed in fresh samples [10]. Two centuries later, in 1878, these crystalline substances were identified as an organic base [11] and named as “spermine” in 1888 [12]. In 1898, the role of spermine in inhibiting bacterial growth and curing various diseases was reported [13]. The structure of polyamines was not known until 1924 when Rosenheim synthesized putrescine (C_4_H_12_N_2_), spermine (C_10_H_26_N_4_), and the related base spermidine (C_7_H_19_N_3_) for the first time [14]. In 1952, the occurrence of the di-amine putrescine was reported in potassium-deficient barley plants [15]. To our knowledge, the putrescine conjugate feruloyl-putrescine (*N*-(4-aminobutyl)-4-hydroxy-3-methoxy-cinnamamide; originally called subaphylline) was the first PAs compound isolated and identified from grapefruit (*Citrus* × *paradisi*) leaves and juice [16]. In the same year (1965), feruloyl-putrescine was also reported in some varieties of sweet orange (including Hamlin, Navel, Pineapple, and Valencia) and grapefruit (including Duncan, Marsh, and Ruby red), but not in Dancy tangerine, lemons, limes, and ‘Cleopatra’ mandarin [16].

PAs are ubiquitous biogenic amines that are found in all living organisms from bacteria to Archaea, and Eukaryotes including plants and animals [3,5,17,18]. For instance, PAs and their biosynthetic genes were reported in phytopathogenic fungi [19,20] such as the Ascomycota fungi *Sclerotinia sclerotiorum*, *Fusarium* spp., *Nectria hematococa*, *Mycosphaerella* spp. and *Magnaporthe grisea* and the Basidiomycota fungi *Puccinia graminis tritici*, *P. triticina*, and *Ustilago maydis* [19,20]. In addition, PAs and their biosynthetic genes were reported in phytopathogenic fungi-related Chromista organisms (formally known as oomycetes or stramenopiles) such as *Phytophthora infestans*, *P. ramorum*, and *P. sojae* [19]. Moreover, PAs were reported in bacteria from families Aeromonadaceae, Halomonadaceae, Pasteurellaceae, Pseudomonadaceae, and Vibrionaceae, and other related genera of the gamma subclass of the Proteobacteria [21,22,23,24], although some bacterial genera do not synthesize them [25]. Additionally, PAs were found in bacterial viruses (bacteriophage) [26,27]. 

**Figure 1:**
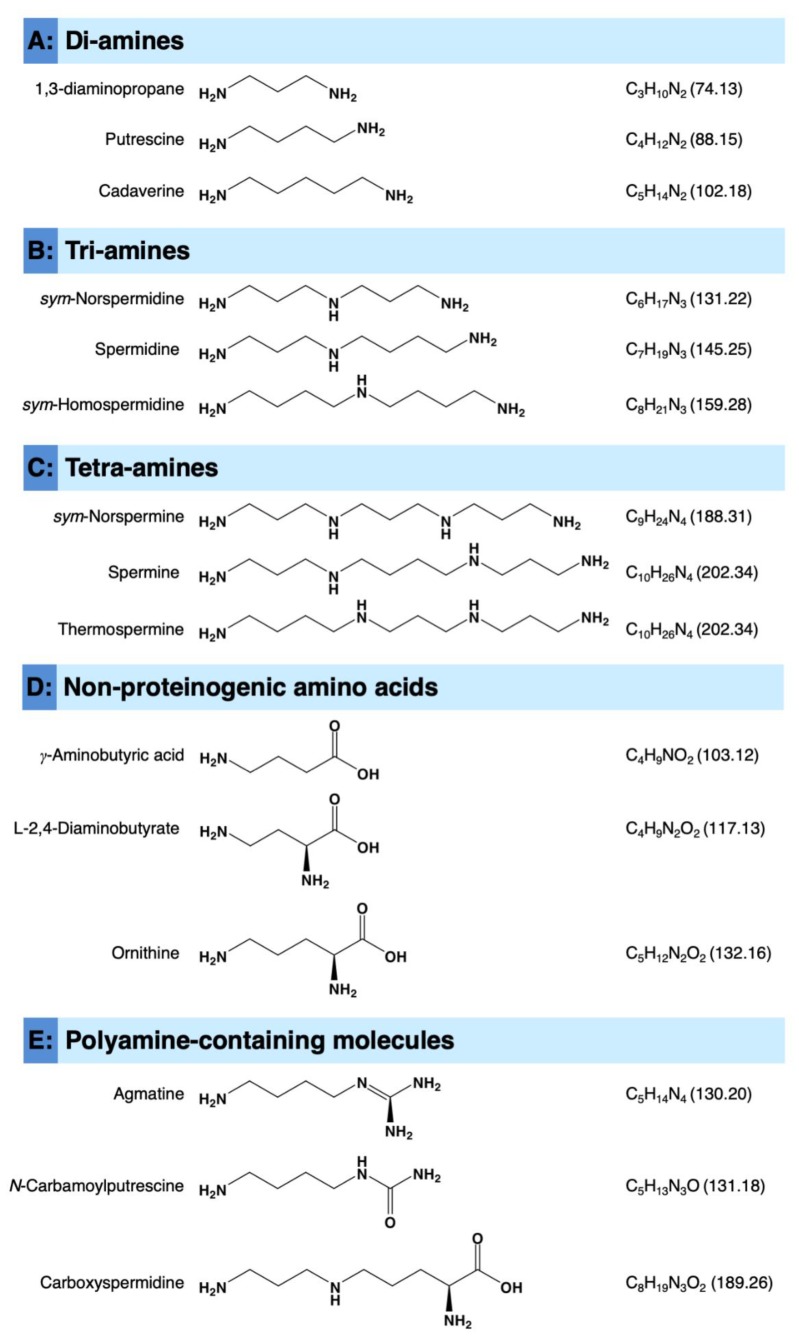
Chemical structures of the polyamines (PAs) and other nitrogen-containing molecules discussed throughout this review. (**A**) Diamines, (**B**) Tri-amines, (**C**) Tetra-amines, (**D**) Non-proteinogenic amino acids, and (**E**) Polyamine-containing molecules. Molecular weight/molar mass (g.mol^−1^) is mentioned between parentheses beside the chemical formula of each compound.

In higher plants, putrescine (di-amine), spermidine (tri-amine), and spermine (tetra-amine) (Figure 1) are the major ubiquitously found PAs [28,29,30]. However, other PAs such as the di-amines 1,3-diaminopropane (C_3_H_10_N_2_) and cadaverine (C_5_H_14_N_2_) (Figure 1A); the tri-amines *sym*-norspermidine (C_6_H_17_N_3_) and *sym*-homospermidine (C_8_H_21_N_3_) (Figure 1B); and the tetra-amines *sym*-norspermine (C_9_H_24_N_4_) and thermospermine (C_10_H_26_N_4_) (Figure 1C) and longer-chain or branched polyamines are less widely distributed [30].

The genus *Citrus* (family *Rutaceae*) is considered one of the most widely grown fruit crops worldwide [31]. *Citrus* spp. can grow within 35–40 ° north and south latitude [31,32]. Therefore, they consider being native to the tropical and subtropical areas [31,32]. Previously, several metabolic signaling pathways were reported to be involved in physiological functions in citrus plants, including amino acids, organic acids, fatty acids, phytohormones, polyamines, and other secondary metabolites.

In citrus, PAs play an important role(s) in plant growth, development, and other physiological processes. These physiological processes include embryogenesis [33,34]; root system formation, morphology, and architecture [35,36,37,38,39,40,41,42]; plant growth and shoot system architecture [35,37,43,44,45,46,47]; inflorescence, flowering and flowering-associated events [48,49,50,51,52,53,54]; fruit set, development, and quality [55,56]; stomatal closure and gas-exchange [39,47,57,58,59]; and photosynthesis and chlorophyll fluorescence [37,40,45,47,60,61,62]. However, the molecular mechanisms behind these roles remain ambiguous. Accordingly, in the present review, we discuss the biosynthesis of PAs in citrus plants, with an emphasis on the recent advances in identifying and characterizing PAs-biosynthetic genes and other upstream regulatory genes involved in transcriptional regulation of PAs metabolism. In addition, we are going to discuss the recent metabolic, genetic, and molecular evidence illustrating the roles of PAs in citrus physiology including embryogenesis, root and shoot systems architecture, flowering, fruit set, gas-exchange, and photosynthesis. 

## 2. Biosynthesis of Polyamines (PAs) in Citrus Plants

**Figure 2:**
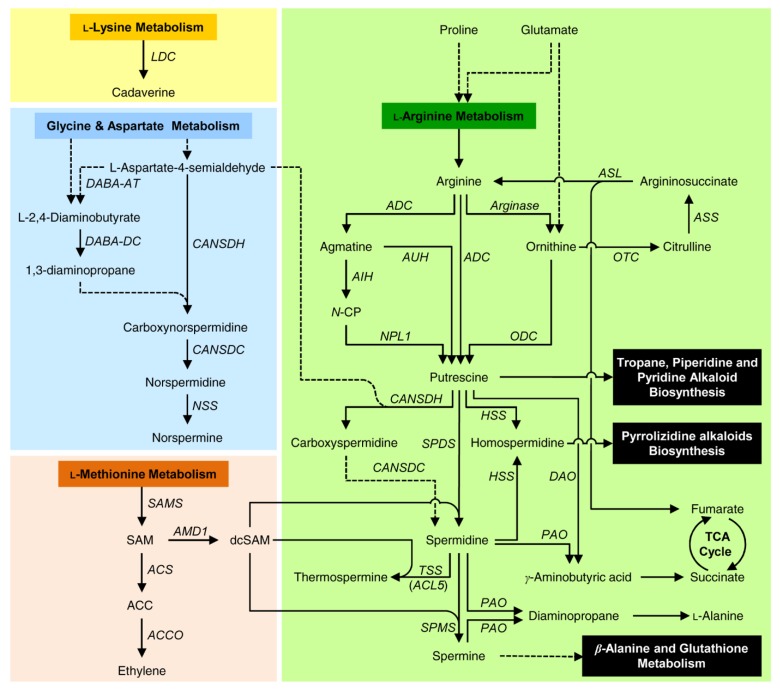
Key steps in the polyamines (PAs) biosynthesis pathway and its associated genes in citrus. Genes are mentioned in italic. Abbreviations: ACC: 1-Aminocyclopropane-1-carboxylic acid, *ACCO*: 1-Aminocyclopropane-1-carboxylic acid oxidase, *ACS*: 1-Aminocyclopropane-1-carboxylic acid synthase, *AIH*: Agmatine deiminase/iminohydrolase, *AUH*: Agmatine ureohydrolase (also known as agmatinase), *ADC*: Arginine decarboxylase, *ASL*: Argininosuccinate lyase (also known as argininosuccinase), ASS: Argininosuccinate synthase, *CANSDC*: Carboxynorspermidine/carboxyspermidine decarboxylase, *CANSDH*: Carboxynorspermidine/carboxyspermidine dehydrogenase (also known as Carboxynorspermidine/ carboxyspermidine synthase), dcSAM: decarboxylated S-adenosylmethionine, *DAO*: Diamine oxidase, *DABA-DC*: Diaminobutyrate decarboxylase (also known as L-2,4-diaminobutyrate decarboxylase), *DABA-AT*: Diaminobutyrate-2-oxoglutarate transaminase (also known as L-2,4-diaminobutyrate:2-Oxoglutarate 4-aminotransferase), *HSS*: Homospermidine synthase, *LDC*: Lysine decarboxylase, N-CP: N-carbamoylputrescine, *NPL1*: N-Carbamoylputrescine amidohydrolase (also known as N-carbamoylputrescine amidase (*CPA*)), *NSS*: Norspermine synthase (also known as long-chain polyamine synthase), *ODC*: Ornithine decarboxylase, *OTC*: Ornithine transcarbamylase, *PAO*: Polyamine oxidase, SAM: S-Adenosyl methionine, *AMD1*: S-Adenosyl-methionine decarboxylase (also known as *SAMDC*), *SAMS*: S-Adenosyl-methionine synthase, *SPDS*: Spermidine synthase, *SPMS*: Spermine synthase, and *TSS*: Thermospermine synthase encoded by ACAULIS5 (*ACL5*).

The biosynthetic pathway of PAs has been extensively studied in all the kingdoms of living organisms, from Bacteria to Archaea and Eukaryotes [29,63,64,65,66,67,68,69,70]. However, our knowledge about this pathway and the genes involved in citrus is still limited. In general, PAs are synthesized in citrus plants from the proteinogenic amino acid l-Arginine through two different arginine-dependent routes. The first route is similar to that of animals and fungi, where l-arginine is converted by mitochondrial arginase (EC 3.5.3.1) to the non-proteinogenic amino acid ornithine, which is then decarboxylated by ornithine decarboxylase (ODC; EC 4.1.1.17) to form the diamine putrescine (Figure 2). Moreover, citrus plants could use an additional route involving arginine decarboxylation by arginine decarboxylase (ADC; EC 4.1.1.19) to produce agmatine, which catalyze to N-carbamoylputrescine (N-CP) then to putrescine by agmatine iminohydrolase (AIH, also known as agmatine deiminase; EC 3.5.3.12) and N-carbamoylputrescine amidohydrolase (NPL1, also known as N-carbamoylputrescine amidase (CPA); EC 3.5.1.53), respectively. In addition, l-arginine and agmatine could contribute directly toward the production of putrescine via the catalytic actions of ADC and agmatine ureohydrolase (AUH, also known as agmatinase; EC 3.5.3.11), respectively (Figure 2). 

Interestingly, the enzymatic activities and transcriptomic analyses of both ODC and ADC (the rate-limiting enzymes in PAs biosynthesis) were reported previously from several citrus varieties such as ‘Murcott’ mandarin (*Citrus reticulata*) [71], Valencia sweet orange (*C. sinensis*) [34], and *C. clementina* [56]. Additionally, phylogenetic comparison of citrus loci of PAs biosynthetic genes with those from *Arabidopsis thaliana* showed that *ADC* from citrus was presented as one locus in comparison with two loci from *A. thaliana* [72]. In contrast, *ODC*, which is not present in *A. thaliana* [73], was identified in *Citrus* and presented a similar number of loci (only one locus) as those described in *Oryza sativa*, and *Glycine max* [72]. 

The presence of the active forms of both *ADC* and *ODC* was previously reported in different citrus species under biotic and abiotic stressors [46,72,74,75]. Taken together, the presence of both enzymes indicates that citrus could have two alternative routes to generate putrescine from l-arginine. These two routes could be activated differently upon different stressors. However, further studies are required to identify and explain the physiological function(s) of each route. 

Once putrescine is produced, it is then converted into the tri-amine spermidine by spermidine synthase (SPDS; EC 2.5.1.16), with the addition of an aminopropyl moiety donated by decarboxylated *S*-adenosylmethionine (dcSAM). dcSAM is synthesized from the proteinogenic amino acid l-methionine via two sequential reactions that catalyze l-methionine to S-adenosyl methionine (SAM) by S-adenosyl-methionine synthase (SAMS, also known as methionine adenosyltransferase; EC 2.5.1.6), then converted to dcSAM by S-adenosyl-methionine decarboxylase (AMD1, also known as SAMDC; EC 4.1.1.50) (Figure 2). Moreover, putrescine could be converted to spermidine via carboxyspermidine, as an intermediate, by carboxynorspermidine dehydrogenase (CANSDH, also known as carboxynorspermidine/carboxyspermidine synthase; EC 1.5.1.43) and carboxynorspermidine decarboxylase (CANSD*C*; EC 4.1.1.96), respectively, with the addition of an amide group donated by L-aspartate-4-semialdehyde from glycine metabolism pathway (Figure 2). Both putrescine and spermidine support the production of the tri-amine homospermidine using homospermidine synthase (HSS; EC 2.5.1.44). Finally, spermidine is then converted into the tetra-amines spermine or thermospermine by the incorporation of another aminopropyl group (also from dcSAM) using spermine synthase (SPMS; EC 2.5.1.22) and thermospermine synthase (TSS, encoded by a gene named ACAULIS5 (ACL5); EC 2.5.1.79), respectively (Figure 2). Originally, it was thought that two genes (*SPMS* and *ACL5*) both encode for SPMS enzyme in *A. thaliana* [76,77]. However, *ACL5*, from *Thalassiosira pseudonana* and *A. thaliana*, was reported to catalyze the conversion of spermidine to thermospermine but not to spermine [78]. Intriguingly, *ACL5* genes have been expressed during parthenocarpic fruit development in *C. clementina* [56].

Noteworthy, phylogenetic analysis showed that SPDS from citrus is presented as one locus only in comparison with two loci from A. thaliana [72]. On the other hand, AMD1, SPMS, TSS (also known as ACL5), NLP1, and AIH enzymes were identified in Citrus and presented a similar number of loci as those described in A. thaliana [72]. Sweet orange (*C. sinensis*) constitutively overexpressing *SPDS1* from apple *resulted in* significantly higher endogenous polyamine content, particularly the free spermine [79]. In addition, the flux of radioactivity from l-[3,4-^14^C] methionine into [^14^C]spermidine was greatly induced when the enzyme 1-aminocyclopropane-1-carboxylic acid synthase (ACS (EC 4.4.1.14), responsible for the conversion of SAM to ACC) was inhibited by aminoethoxyvinylglycine (AVG) in aged peel discs of orange (*C. sinensis*) fruit [80]. While AVG reduced the flux of radioactivity to ACC, it increased the label in SAM [80]. The accumulated SAM could contribute directly to increase the dcSAM levels, which subsequently enhance the spermidine and spermine synthesis. Furthermore, AMD1 (also known as CsSAMDC) was previously cloned and biochemically identified from the navel orange (*C. sinensis*). Taken together, this provides strong evidence for the physiological link between dcSAM, produced by the catalytic actions of AMD1 (also known as CsSAMDC), and the biosynthesis of tri- and tetra-amines. 

Furthermore, some other PAs could be derived from other proteinogenic amino acids rather than l-arginine. For instance, the diamine cadaverine is synthesized from l-lysine by the catalytic actions of lysine decarboxylase (LDC; EC 4.1.1.18) (Figure 2). High levels of cadaverine were recorded in immature seeds of citrus species [81,82,83,84]. For example, cadaverine covalently bound in the perchloric acid-insoluble fraction of flesh and peel of Brasiliano N.L. 92 (*C. sinensis*) unripe and ripe fruits [82]. Recently, the free, soluble-conjugated, and insoluble-bound forms of cadaverine were reported in Kinnow mandarin (*C. nobilis* × *C. deliciosa*) plants grafted on diploid (2x) and double-diploid (4x) rootstocks of *P. trifoliata*, *C. reshni*, and *C. limonia* [84]. Cadaverine and its biosynthetic gene (*LDC*) may be used as biochemical markers of potassium (K) deficiency [83], as well as playing a key role in chromium-tolerance in Kinnow mandarin [84]. However, to our knowledge, the LDC of citrus is not yet cloned.

Glycine and l-aspartate metabolism pathways could be involved in the biosynthesis of PAs, particularly the tri-amine norspermidine. Briefly, both glycine and l-aspartate metabolism pathways input to produce L-aspartate-4-semialdehyde which donates a carboxy-aminopropyl group to propane-1,3-diamine to form carboxynorspermidine by CANSDH, then Norspermidine and Norspermine by CANSDC and Norspermine synthase (NSS, also known as long-chain polyamine synthase; EC 2.5.1.126), respectively (Figure 2). Norspermidine could be produced from L-aspartate-4-semialdehyde via the production of L-2,4-diaminobutyrate by L-2,4-diaminobutyrate:2-oxoglutarate 4-aminotransferase (DABA-AT, also known as diaminobutyrate-2-oxoglutarate transaminase; EC 2.6.1.76), then the conversion to 1,3-diaminopropane by diaminobutyrate decarboxylase (DABA-DC, also known as L-2,4-diaminobutyrate decarboxylase; EC 4.1.1.86). 

## 3. Physiological Roles of PAs in Citrus

During the last two decades, multitudinous studies have highlighted the regulatory roles of PAs in different physiological processes of plant growth and development (for details see reviews by [85,86,87,88,89,90,91,92]. Most, if not all, of these studies demonstrated that PAs have many similarities with the five classic groups of phytohormones [93,94]. Therefore, PAs have been proposed recently as a new class of plant growth regulators (PGRs) [94,95]. However, plant physiologists have not yet given PAs the status of phytohormones unequivocally, perhaps because PAs differ from phytohormones in two respects; (I) the concentration of PAs in plant tissue is much higher compared to phytohormones (mM vs μM); and (II) in contrast with most phytohormones, PAs are poorly translocated. 

PAs play vital multi-regulatory roles in most, if not all, growth- and development-related processes in citrus plants. However, our knowledge about these roles is still limited, and in fact, many roles have not yet been discovered. These processes including somatic embryogenesis [33,34,96], seed germination [97], plant growth (including biomass production, plant height, fresh and dry weight, and number of leaves/plant) [35,36,44,45,47], root morphogenesis and architecture [35,36,37,38,39,40,41,42], photosynthesis and photosynthetic pigments [35,37,45,60], and reproductive development during flowering [49,50,51,88] and fruit set [55,71,88,98]. In the next paragraphs, we will discuss the multi-regulatory roles of PAs in citrus plants.

## 4. Role of PAs in Somatic Embryogenesis of Citrus Plants 

Somatic embryogenesis is a complex multi-stage process to regenerate new plants in vitro through the tissue culture technique. During the somatic embryogenesis, differentiated somatic cells are used to develop cellular structures similar to zygotic embryos, subsequently generating whole plant bodies. Somatic embryogenesis involves several morphological, physiological, transcriptional, and biochemical changes [99,100]. In vitro, somatic embryogenesis, and plant regeneration, in general, are controlled by exogenous supplementation of plant hormones [101]. Auxin and cytokinin are the main phytohormones involved in this process. The balance between auxin and cytokinin regulates the developmental fate of regenerating plant organs [101].

Although the role of auxin and/or cytokinin in somatic embryogenesis in citrus was intensively studied previously [102,103,104,105,106,107,108], their roles are still controversial. Some scientists suggest that auxins and their synthetic analogs (such as indole-3-acetic acid (IAA), α-naphthalene acetic acid (α-NAA), and 2,4-dichlorophenoxyacetic acid (2,4-D)) strongly inhibit the somatic embryogenesis [103,105] and somatic embryogenesis could be enhanced by lowering the auxin status and using the inhibitors of auxin synthesis [103,104]. On the contrary, others believe that increasing the level of auxins such as 2,4-D and NAA in tissue culture media was effective in callus induction in somatic embryogenesis [107,108].

In contrast with the controversial role of auxins, evidence suggests that PAs are positively associated with somatic embryogenesis in citrus [33,34,96], although the exact mechanisms through which they exert an effect have not yet been fully elucidated. Liu et al. (2005) analyzed the changes in polyamine levels during somatic embryogenesis using a long-term (8 years) sub-cultured calli of *C. sinensis* cv. Valencia. Their findings showed that endogenous PAs content of embryogenic calli was significantly higher than those in non-embryogenic calli, and the embryogenetic capability was positively correlated to the endogenous levels of PAs [33]. Furthermore, putrescine reached its highest level at an early stage of differentiation, while spermidine and spermine increased and reached their peak at the globular embryo stage and at a later stage of globular embryo development, respectively [33]. 

In addition, Wu et al. (2009) noticed that culturing the embryogenic callus of *C. sinensis* on the embryo-inducing medium (EIM) containing glycerol produced a large number of embryos, which were positively correlated with the endogenous levels of PAs (spermidine and spermine) during the first 20 days of culture, coincident with abundant somatic embryogenesis [34]. Moreover, the expression levels of the four key genes involved in PAs biosynthesis (*CsADC*, *CsODC*, *CsSPDS*, and *CsSPMS*) were higher in cultures on EIM, and that their transcriptional levels were increased with the developmental stages of the embryos [34]. Likewise, the induction in *CsADC* activity [33] and *CsSPDS* gene expression [96] were positively correlated to the endogenous levels of PAs during somatic embryogenesis in citrus plants. Similar results were found in other plant species such as *Pinus radiata* [109], *Panax ginseng* [110], and *Picea rubens* [111]. Additionally, overexpression of polyamine oxidase 4 of citrus (*CsPAO4*; a key gene in PAs catabolism that uses both spermidine and spermine as substrates for terminal catabolism) in transgenic lines of *Nicotiana tabacum* displayed higher PAO activity, lower spermidine, and spermine levels, and down-regulation of late embryogenesis abundant protein gene (*NtLEA5*) compared with wild-type tobacco plants [112]. 

Moreover, the addition of α-difluoromethylornithine (DFMO; an inhibitor of PAs biosynthesis) to the EIM inhibited the somatic embryogenesis remarkably, concurrent with a significant reduction of endogenous PAs levels [34]. However, exogenous supplementation of putrescine with [34] or without DFMO [33] enhanced the embryogenesis frequency and increased the endogenous PAs levels [33,34]. All of these, collectively, demonstrate that PAs play a key role in the regulation of somatic embryogenesis in citrus plants. The regulatory role of PAs in somatic embryogenesis could be due to the interactions between PAs and nitric oxide (NO). Previous studies showed that exogenous supplementation of PAs to the culture media of several plant species such as *Pinus taeda* (Family: Pinaceae), *Ocotea catharinensis* (Family: Lauraceae), and *Araucaria angustifolia* (Family: Araucariaceae), altered the endogenous levels of NO in somatic embryos [113,114,115,116], which support our suggestion that interactions between PAs and NO might play a key role in somatic embryogenesis (for more details see the review of [117]. Another reason behind the regulatory role of PAs in somatic embryogenesis could be due to the suggestion that PAs act as growth regulators [94,95,118,119] that function as secondary messengers for phytohormones, particularly auxins, within the plant cells [120,121].

## 5. Role of PAs in the Root System Architecture of Citrus Plants

Plant roots play a vital role in the uptake of nutrients and water, anchoring and mechanical support of the plant, storage functions, and interaction with soil microbial flora and abiotic factors [122]. The root system architecture is a key determinant of nutrient- and water-use efficiency in plants. Root system architecture and development are controlled by phytohormonal, metabolic, and environmental cues that might act on genetically controlled developmental processes. From a metabolic point of view, root architecture and development involve not only the five classic phytohormone groups but also other PGRs, such as PAs [87]. 

**Figure 3:**
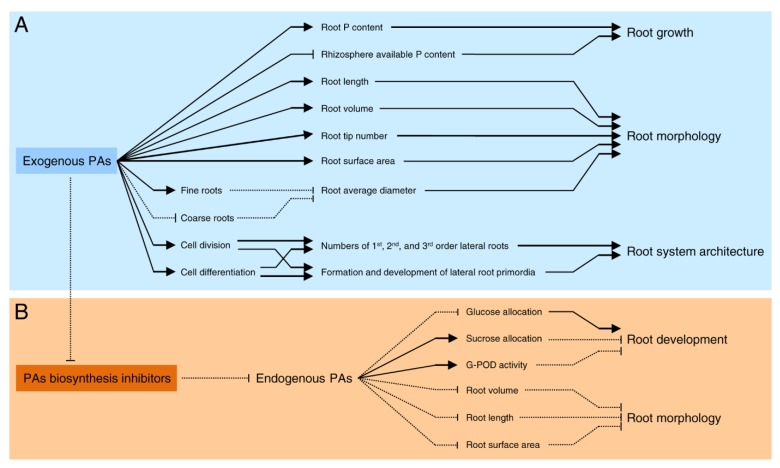
Hypothetical model of the effect of (**A**) exogenous polyamines (PAs) and (**B**) PAs biosynthesis inhibitors on root growth, development, morphology and root system architecture of citrus plants. Solid lines with arrows signify positive reaction, while round-dotted lines with bar-ends indicate negative interaction. For more details, see the main text.

In citrus, several studies have outlined the role of PAs in root formation, morphology, and architecture [35,36,37,38,39,40,41,42] (Figure 3). In general, modifications of the endogenous levels of PAs by chemical inhibitors [36,39], by mutation or gene manipulation [74,112], or by exogenous supplementation [35,36,37,40,41] can directly affect the root morphogenesis, development, and subsequent architecture. The exogenous supplementation of PAs increased root length, root projected surface area, root volume and root tip number in Canton lemon (*C. limonia*) [35,40], *C. tangerine* seedlings [41], and trifoliate orange (*P. trifoliata*) seedlings [37]. PAs did not improve the root length of all diameters, but only the diameters below 0.4 mm (more precisely, diameters 0.2-0.4 mm) [35]. Nevertheless, PAs treatment did not affect the root average diameter [37], but sometimes it was decreased in PAs-treated plants [35,40]. The reduction in the average root diameter might be due to the increase in fine roots (%) and the decrease in coarse roots [40]. 

Likewise, the addition of PAs to a medium containing Murashige and Skoog salts significantly improved the root formation and growth of two sweet orange cultivars (*C. sinensis* ‘Pineapple’ and ‘Pêra’) in a concentration-based manner [36]. Furthermore, PAs supplementation increased the numbers of first, second, and third-order lateral roots in PAs-treated plants [35,37] indicating that PAs regulate the root system architecture via controlling the formation and development of lateral root primordia. These effects may be associated with the well-known role of PAs in the regulation of cell division and differentiation in the root apex, and during lateral and adventitious root formation [87]. Likewise, PAs supplementation significantly decreased the available phosphorus content of rhizosphere, while it notably induced phosphorus level in the roots of treated plants [41], which concluded that exogenous PAs could stimulate root morphology, via controlling root phosphorus level, which could result in enhanced growth performance. 

Moreover, the application of DFMO significantly inhibited root development (including total length, total projected area, total surface area, and total volume) of *P. trifoliata* seedlings [37] and also inhibited root formation and growth of *C. sinensis* [36]. DFMO inhibited the allocation of glucose but stimulated both the allocation of sucrose to roots and guaiacol peroxidase (G-POD; negatively related to root development) activity in roots [37]. Interestingly, the negative effects of DFMO on citrus roots were reversed completely, or at least partially, by the application of putrescine to the DFMO-treated plants [36,37]. Together, these findings indicated that carbohydrate allocation might be associated with endogenous PAs regulation of root development in citrus seedlings. Furthermore, AVG and AgNO_3_ inhibited in vitro rooting in sweet orange (*C. sinensis*), indicating that the ethylene biosynthesis pathway was also required for rhizogenesis in citrus [36]. The relationship between the ethylene biosynthesis pathway and PAs biosynthesis pathway was discussed in the previous section (see the above section entitled “Biosynthesis of PAs in citrus plants”).

## 6. Role of PAs in the Plant Growth and Shoot System Architecture of Citrus Plants

Plant architecture could be defined as the three-dimensional organization of the plant body. For the shoot system (aerial parts), this includes the branching pattern, as well as the size, shape, and position of leaves and flower organs. The shoot system architecture is controlled by several environmental factors and endogenous signals such as phytohormones [123,124,125,126] as well as growth regulators such as PAs [89,94,95,118,119,127,128,129]

In higher plants, the plant architecture and growth of axillary buds are initially controlled by auxins and cytokinins. Auxins were previously reported as a direct regulator of plant architecture. Auxins are synthesized in the apical meristems, are translocated basipetally (downward through the phloem), and suppress axillary bud outgrowth, causing a phenomenon known as apical dominance [123,124,130]. Whereas, cytokinins are synthesized in the root tip meristems, travel acropetally (upward through the xylem), and promote/stimulate axillary bud outgrowth [123,124]. Therefore, cytokinin functions as a second signaling molecule that mediates the role of auxin in the regulation of apical dominance [131]. Both auxins and cytokinins interact antagonistically, it has been reported that kind of organogenesis is controlled by relative concentrations of auxins and cytokinins [123,124,125,126].

In addition to auxins and cytokinins, there is accumulating evidence that PAs also play an important regulatory role in plant growth and shoot system architecture, as they do in other horticultural crops [132,133]. PAs and/or PA-conjugates have been analyzed with respect to plant growth and shoot system architecture in intact plants and in in vitro systems. Most previous studies on intact plants have reported a positive correlation between citrus growth and the appearance of a particular class of PAs compounds [35,37,43,44,45,46,47].

For example, the exogenous PAs supplementation beneficially affected the seedling growth of Canton lemon (*C. limonia*), represented by increased biomass, increased shoot and root fresh weight to different extents [35]. Likewise, in the presence of the arbuscular mycorrhizal fungus, *Glomus versiforme*, the application of putrescine and spermine increased the total dry weight of trifoliate orange (*P. trifoliata*) seedlings [44]. Similarly, exogenously applied spermidine by addition to the saline Hoagland nutrient solution (HNS) or via weekly sprays enhanced the accumulation of total spermidine and spermine contents and improved the leaf number of Troyer citrange (*P. trifoliata* × *C. sinensis*) [45]. Furthermore, the expression of PAs biosynthetic (*ADC*, *SAMDC*, *SPDS*, and *SPMS*) and catabolic (*DAO* and *PAO*) genes was significantly up-regulated by PAs [46].

Correspondingly, exogenous application of putrescine significantly increased plant height, stem diameter, leaf number per plant, and shoot, and total dry weights of trifoliate orange (*P. trifoliata*) seedlings compared with the non-treated plants [37]. By contrast, the application of the PAs inhibitor, DFMO, significantly inhibited these metrics; however, the inhibitory effect was partly or completely reversed by exogenous putrescine application to the DFMO-treated plants [37]. Furthermore, the foliar application of 0.5 mM spermidine enhanced growth parameters of Iranian mandarin (*C. reticulata × C. limetta* cv. Bakraii), as represented by leaf number, shoot length, total plant dry weight, and total plant fresh weight [47].

**Figure 4:**
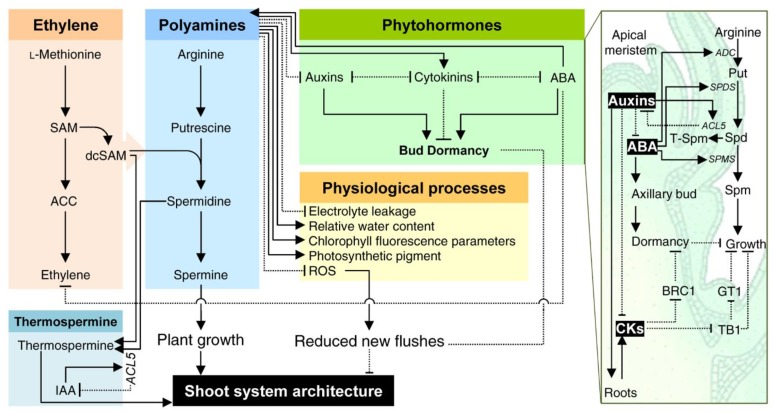
Hypothetical model of the integration of polyamines (PAs) with the major groups of phytohormones in the regulation of the shoot system architecture of citrus plants. Solid lines with arrows signify positive reaction, while round-dotted lines with bar-ends indicate negative interaction. Abbreviations: ABA: Abscisic acid, ACC: 1-Aminocyclopropane-1-carboxylic acid, *ADC*: Arginine decarboxylase, CKs: Cytokinins, dcSAM: decarboxylated S-adenosylmethionine, IAA: indole-3-acetic acid, Put: Putrescine, ROS: Reactive oxygen species, SAM: S-Adenosyl methionine, Spd: Spermidine, *SPDS*: Spermidine synthase, Spm: Spermidine, *SPMS*: *Spermine synthase*, and T-Spm: thermospermine. For more details, see the main text.

It was suggested that the enhancement of citrus growth might be due to the fact that PAs acts as a hormonal second-messengers of cell proliferation and differentiation in many processes [37,134], or regulates the plant sensitivity to auxins/cytokinins ratio [131,135]. In *A. thaliana*, PA-deficient mutants were hyposensitive to auxin and hypersensitive to cytokinin [131]. PAs might play a key regulatory role in citrus morphology and shoot system architecture as well, through controlling the plant response(s) to the auxin/cytokinin ratio. Therefore, we suggest that the interaction between PAs and auxin/cytokinin ratio in citrus tissues determines the initiation of shoot buds (Figure 4). However, the mechanism(s) of how PAs change the sensitivity of citrus plants to auxins and cytokinins is poorly understood and more investigations should be pursued in this area. 

In addition, the regulatory role of PAs in citrus growth and shoot system architecture could be modulated by other phytohormones such as abscisic acid (ABA) (Figure 4). Exogenously applied ABA increased the endogenous content of putrescine and spermine in *Populus popularis* (drought-tolerant genotype) compared with the drought-sensitive poplar genotype [136] and ABA-treated wheat (*Triticum aestivum*) [137]. However, the spermidine levels were decreased after ABA treatment [137], or at least were unaffected [136]. Moreover, the transcript levels of the major PAs biosynthetic genes (*ADC2*, *SPDS1*, and *SPMS*) were up-regulated in wild type plants of *A. thaliana* subjected to water stress; however, these genes were impaired in the ABA-deficient (*aba2*-*3*) and ABA-insensitive (*abi1*-*1*) mutants [138]. Likewise, the transcript level and activity of *ADC2* were reduced in the knockout mutant of the *NCED3* gene (*nc3-2*) in *A. thaliana* [139]. These findings indicated that ABA modulates PAs metabolism at the transcription level by up-regulating the PAs-biosynthetic genes [138,139].

Additionally, the positive effects of PAs in improving the growth of citrus plants could be due to the reduction in electrolyte leakage, increasing the relative water content, chlorophyll fluorescence parameters, photosynthetic pigment concentration [47], as well as reprograming the oxidative and nitrosative status and enhancing the activities of antioxidant enzymes [46,47].

Moreover, PAs might play a key role in the regulation of plant vasculature. For instance, relatively high contents of putrescine, spermidine, and spermine were detected in xylem exudates of citrus and some other species [140]. Furthermore, thermospermine was detected in *Arabidopsis* wild-type, but not *acl5* mutants [141]. The absence of thermospermine in *acl5* mutants caused stem growth imperfection, which partially rescued by exogenous supplementation of thermospermine [141]. Several genetic and molecular evidence suggest that thermospermine might play a fundamental role in preventing maturation and death of the xylem vessel elements [65]. Nevertheless, the role of PAs and their biosynthetic genes in citrus vasculature is poorly studied and more investigations should be pursued in this area.

Previously, the ectopic expression of the *spermidine synthase* gene from apple (*MdSPDS1*) into sweet orange (*C. sinensis*) via *Agrobacterium*-mediated transformation of embryogenic calluses resulted in enhanced growth, higher endogenous PAs levels, and reduced canker susceptibility on transgenic lines compared with wild-type plants [79]. Moreover, it has been shown recently that thermospermine is involved in growth and gene expression of PAs-associated enzymes in rice seedlings [142]. Likewise, it was reported that both thermospermine synthase (*ACL5*) and diamine oxidase (*DAO*) genes are required for zygotic embryogenesis and vascular development in scots pine (*Pinus sylvestris*) [143]. Collectively, these findings indicate that thermospermine is critical for proper vascular development and xylem cell specification. However, the molecular mechanism(s) behind this role remains ambiguous and it is poorly understood how thermospermine homeostasis controlled in the xylem. A negative auxin-dependent feedback loop mechanism was reported previously to control the thermospermine levels in stem xylem tissues when *ACAULIS5* (also known as *ACL5*) was cloned from hybrid aspen (*Populus tremula* × *P. tremuloides*) and black cottonwood (*P. trichocarpa*) [144]. Briefly, the over-expression of the *ACL5* in *Populus* resulted in the up-regulation of *ACL5* expression and enhanced thermospermine accumulation, but negatively influenced the accumulation of indole-3-acetic acid (IAA). On the other hand, exogenous auxin supplementation enhanced *ACL5* expression. Taken together, these results support the suggestion that thermospermine plays a key role in vascular development particularly and shoot system architecture in general (Figure 4) 

## 7. Role of PAs in the Flowering and Inflorescence of Citrus Plants

The juvenile phase (also known as the vegetative phase) of *Citrus* species is relatively long, taking 2-5 years to reach maturity period (also known as reproductive phase) and produce flowers [145,146]. In the maturity phase, the developed inflorescence may be either leafy or leafless and may carry multiple flowers or only one of them [145,146,147]. Citrus flowering is a complex phenological process and the floral load is affected by the genotype, cultivar, age, and environmental conditions [145,146]. *Citrus* species usually overproduce flowers during the blooming season, leading to a high abscission rate and low fruit set [145]. The coordinated transition from vegetative phase to flowering, or reproductive phase in general, is controlled by a complex genetic network, several external environmental cues (such as photoperiod and temperature, etc.) [145,146,147], as well as by endogenous factors such as phytohormones [148] and PAs [92,93,128].

**Figure 5:**
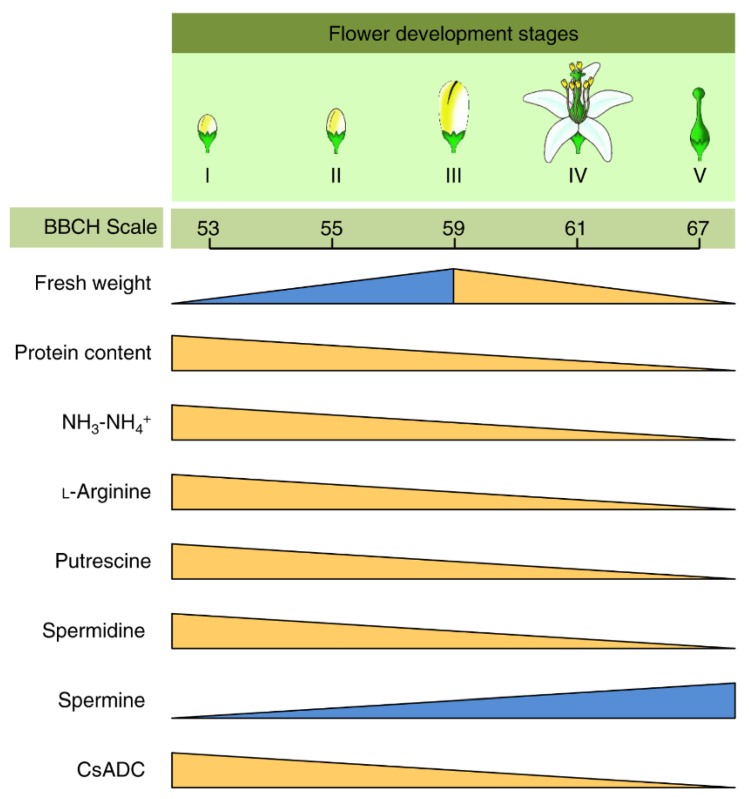
Schematic overview representation of the metabolic behavior of polyamines (PAs), some other nitrogen-containing metabolites, and the major PAs-biosynthetic gene, Arginine decarboxylase (*ADC*), during the floral development of citrus plants. Flower development stages (53, 55, 59, 61, and 67) are corresponding to the phenological growth stages on BBCH-scale [149,150]. Yellow bars/triangles represent the reduction in the metabolite levels or down-regulation in gene expression, while blue bars/triangles represent the increase in the metabolite levels or up-regulation in gene expression. For more details, see the main text.

Several previous studies outlined the close connection between nitrogen metabolism in general, PAs particularly, and the flowering-associated physiological events in citrus plants [48,49,50,51,52,53,54]. For instance, PAs were reported to be involved in the compatible and self-incompatible pollination of citrus [54]. Briefly, the self-pollinated flowers (self-incompatible pollination model) had higher levels of putrescine, spermidine, and sometimes spermine compared with cross-pollinated pistils in the compatible pollination model [54]. In addition, flowering and inflorescence could be induced by alterations in the sink-source relationship of nitrogen metabolism such as PAs. Changes in the PAs levels and some other nitrogen metabolites during floral development in citrus plants with their corresponding phenological growth stages on BBCH-scale [149,150] are summarized in Figure 5. Briefly, NH_3_–NH_4_^+^ content was higher (more than 4-folds) in Stage I (growth stage 53 of BBCH-scale) of inflorescence emergence of ‘Washington’ navel orange (*C. sinensis*), then it consistently decreased through all subsequent stages of flower development until the petal fall at Stage V (growth stage 67 of BBCH-scale) [50,51] (Figure 5). These findings were consistent with the accumulation of NH_3_–NH_4_^+^ in the leaves of ‘Washington’ navel orange during the developing floral buds stage, which was associated with a higher number of flowers after low temperature-induced flowering [48]. Similar results of higher flowering intensity and NH_3_–NH_4_^+^ content were found in commercial groves of ‘Frost Lisbon’ lemon trees (*C. limon*) subjected to drought-induced flowering [48]. Interestingly, the maximum activity of the l-arginine de novo biosynthesis pathway was also observed in Stage I (growth stage 53 of BBCH-scale) flowers (Figure 5) and decreased throughout the flower development stages in the same manner as NH_3_–NH_4_^+^ content [50,51].

Likewise, putrescine and spermidine were higher in the apical flowers of inflorescences initiated in response to eight weeks of low-temperature stress (Stage I flowers) and decreased in parallel with NH_3_–NH_4_^+^ and l-arginine as flowers developed through Stage V (petal fall; growth stage 67 of BBCH-scale), except for spermidine which did not change significantly between Stage IV (growth stage 61 of BBCH-scale) and Stage V (growth stage 67 of BBCH-scale) flowers [49,50]. Studies on the annual cycle of endogenous PAs of two citrus varieties, 32-year-old ‘Washington’ navel orange (*C. sinensis*) grafted on Troyer (*C. sinensis* × *P. trifoliata*) and 8-year-old Miyagawa Satsuma (*C. unshiu*) grafted on trifoliate orange (*P. trifoliata*), showed partially similar results, where putrescine content changed most dramatically during flowering, peaked at full bloom, and decreased rapidly afterward [151]. In contrast, spermine had lower levels in the Stage I flowers, but increased dramatically, reaching its maximum concentration (approximately 7-folds) during Stage IV (flower opening; growth stage 61 of BBCH-scale) [49,50]. The changes in the levels of l-arginine, putrescine, spermidine, and spermine were not due to increased organ weight since the fresh weight followed a different pattern (Figure 5). 

Interestingly, the exogenous foliar treatment with low-biuret urea, at the end of minimal stress treatment that does not usually result in significant flower production (i.e., 4 instead of 8 weeks of low-temperature treatment or deficit irrigation instead of withholding irrigation, treatment), increased the endogenous ammonia content, which was correlated with higher number of floral shoots and flowers per shoot [51,152]. Likewise, foliar application of putrescine (20 mM) and spermidine (10 mM) significantly increased the number of the total flowers per tree, the number, and proportion of leafless inflorescences per tree [153]. This induced flowering was positively correlated with the endogenous levels of spermidine in citrus leaves at the beginning of the induction treatment [52]. Furthermore, the developed apical flowers had higher levels of ammonia and putrescine and exhibited a higher activity of the de novo arginine biosynthetic pathway [51]. However, all these criteria declined in parallel as flowers developed through stage V flowers (petal fall).

Taken together, these findings demonstrate that the floral buds depend on parallel changes in nitrogen metabolism during low temperature-induced flowering and provide strong evidence for the correlation between NH_3_-NH_4_^+^ content, PAs and their precursor (l-arginine), and the flower development in *C. sinensis* [49,50]. Accordingly, two hypotheses were suggested for nitrogen metabolism-based flowering induction. The first hypothesis is that the higher ammonia level during stress resulted in the accumulation of arginine and PAs, which subsequently could induce the cell division required for bud induction. Alternately, the second hypothesis suggests that these physiological changes and the subsequent induction of cell division are pre-requisites to flower initiation in citrus [48].

In other plant models, the relationship between PAs and flower development has been intensively studied using chemical inhibitors of PA biosynthesis pathway. For example, cyclohexylamine (CHA; an inhibitor of spermidine biosynthesis) prevents spermidine accumulation, inhibits floral initiation, and inhibits flowering, without significant effects on vegetative growth, in *N. tabacum* [154] and *Spirodela punctuata* [155], while spermidine supplementation abolished these inhibitory effects and induced flowering [154,155]. Similarly, using methylglyoxal-bis(guanylhydrazone) (MGBG; a specific inhibitor of SAMDC enzyme) inhibits flowering without significant effects on the vegetative growth of *S. punctuata*, whereas exogenous spermidine application reversed these inhibitory effects and induced flowering [155]. Likewise, using dl-α-difluormethylarginine (DFMA; a specific inhibitor of ADC enzyme) prevented floral bud initiation in thin-layer explants of tobacco, while using DFMO (a specific inhibitor of *ODC* enzyme) did not affect the floral bud initiation [156]. On the other hand, using DFMO prevented the subsequent development of newly regenerated floral buds, but not using DFMA [156]. These findings suggested that floral bud initiation requires ADC-derived PAs, while ODC-derived PAs are required for subsequent growth and development of newly regenerated floral buds [156]. Although PA biosynthetic inhibitors proved to be powerful tools for studying the relationship between PAs and flower development, to our knowledge, no studies have been carried out in *Citrus*. 

## 8. Role of PAs in the Fruit Set, Development, and Quality of Citrus Plants

In fruit crops, the yield is directly correlated to the fruit set after pollination. Nevertheless, heavy flowering does not necessarily guarantee a subsequent economic crop of fruit. Poor fruit set either by severe post-bloom drop and/or pre-harvest drop could cause significant losses in yield. Fruit set varies among different fruit crops. For instance, it could reach as high as 90% as in blueberries, or it could be as low as 1% as in sweet oranges [157]. Moreover, in addition to the low fruit set, citrus has a noticeable, if not extensive, post-bloom fruit drop (also known as June drop) compared to a small percentage of fruit drop for blueberries [157]. In citrus, fruit set may be affected by tree genetics (such as variety, rootstock, and cultivar), environmental conditions (before, during, and after bloom), and/or endogenous factor (such as carbohydrate competition, phytohormones homeostasis, and PAs content) [51,55,56,71,88]. Although many comprehensive physiological and biochemical descriptive studies have been carried out to explore the endogenous PAs levels and their roles in the citrus fruit set, the enormous unexplored potential exists in this area of study. 

Furthermore, PAs and their biosynthesis genes might play a key role in parthenocarpic ability and ability to set leafy and leafless inflorescences. For instance, the ovaries of ‘Oronules’ mandarin (a self-incompatible cultivar) had a higher PAs content than ‘Okitsu’ mandarin (a male-sterile cultivar with a high degree of natural parthenocarpy) [55].

**Figure 6:**
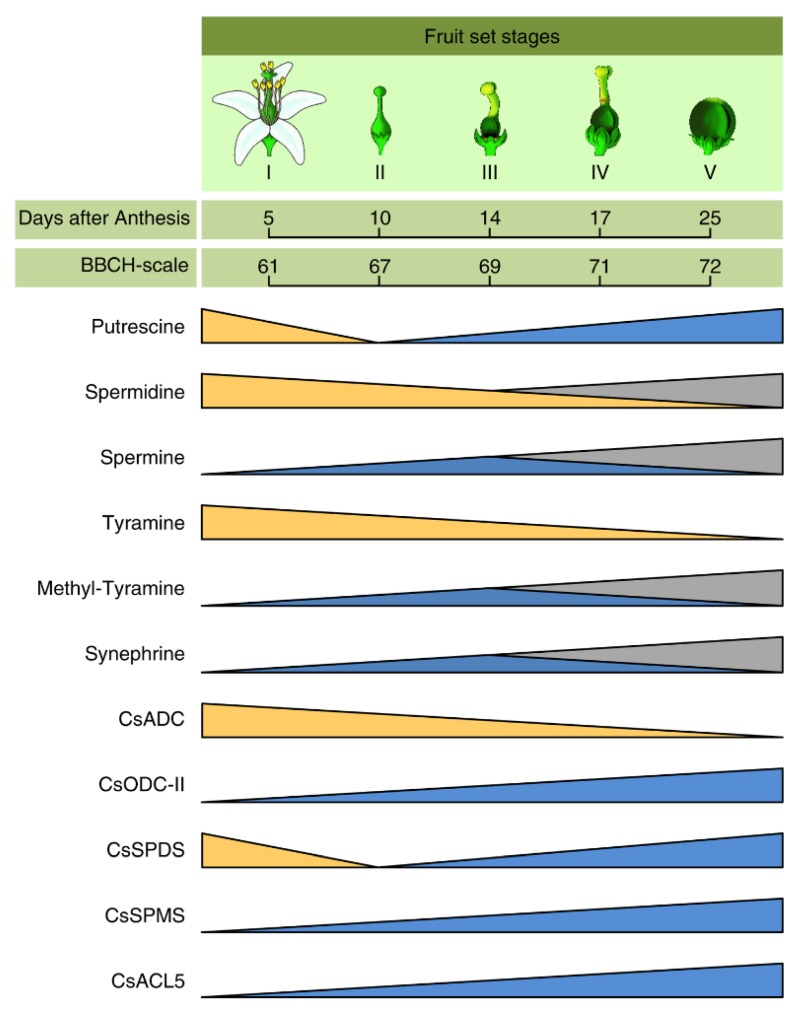
Schematic overview representation of the metabolic behaviors of polyamines (PAs), some other nitrogen-containing metabolites, and the major PAs-biosynthetic genes during the fruit set and development of citrus plants. Fruit set stages (61, 67, 69, 71, and 72) are corresponding to the phenological growth stages on BBCH-scale [149,150]. Yellow bars/triangles represent the reduction in the metabolite levels or down-regulation in gene expression, blue bars/triangles represent the increase in metabolite levels or up-regulation in gene expression, while gray bars/triangles represent the controversial behavior in metabolite levels. For more details, see the main text. Abbreviations: *CsADC*: Arginine decarboxylase, *CsODC*: Ornithine decarboxylase, *CsSPDS*: Spermidine synthase, *CsSPMS*: Spermine synthase, and *CsACL5*: Thermospermine synthase encoded by ACAULIS5.

Changes in the PAs levels, their biosynthetic genes, and some other nitrogen-containing metabolites during the fruit set of citrus plants with their corresponding phenological growth stages of BBCH-scale [149,150] are summarized in Figure 6. In contrast with the role of NH_3_–NH_4_^+^ content in citrus flowering mentioned in the previous section, no distinct relationship between the NH_3_–NH_4_^+^ content and fruiting intensity was observed during induction of both July and February flushes under hot sub-humid tropical climate [158]. Although the major PAs compounds (putrescine, spermidine, and spermine) were detected throughout the fruit set, however, each compound displayed a different profile and relative variations in its content during the different fruit set stages (Figure 6). Briefly, putrescine content transiently dropped during the first 7 or 15 days after anthesis (growth stage 65-67 of BBCH-scale), then it re-established and increased after petal fall through the growth stage 71-72 of the BBCH-scale [55,56]. Likewise, the free and perchloric acid (PCA)-insoluble conjugated putrescine increased in the flesh and peel (flavedo plus albedo) of ‘Brasiliano’ navel orange (*C. sinesis*) during ripening, going from unripe to ripe fruits, and decreased in overripe fruits [82]. However, putrescine levels were varied in the growth stage 71 and 72 of the BBCH-scale depending on the cultivar and the year that experiment was carried out [55,56]. 

Moreover, the exogenous-applications of low-biuret urea, to the leaves of ‘Washington' navel orange during early bloom, increased the endogenous putrescine content and fruit set [51]. Likewise, exogenous application of 0.01 mM aqueous solution of PAs (putrescine, spermidine or spermine) significantly increased the initial fruit set of ‘Blood Red’ sweet orange (*C. sinensis*), with greater effects by spermidine, followed by spermine and putrescine, respectively [98]. Furthermore, putrescine application increased the fruit yield/tree, production of grade-I fruit (%), fruit weight (g), and fruit diameter (cm), whereas exogenous application of the mixture of all three PAs (0.001 mM each) decreased the fruit set and slightly increased the other parameters compared with control plants [98].

Interestingly, the expression levels of putrescine biosynthetic genes, *CsADC* and *CsODC*, showed a contrary behavior. The expression of *CsADC* was decreased throughout the fruit set stages, whereas one of the two *CsODC* paralogs (*CsODC*-II) was up-regulated throughout the fruit set stages [56]. In other words, the expression *CsADC* mirrored the reduction in putrescine levels during the first 7 or 15 days after anthesis, whereas, *CsODC* mirrored the increase in putrescine content in citrus ovaries after petal fall through the growth stage 71-72 of the BBCH-scale [56]. These findings suggest that fruit set initiation requires *ADC*-derived PAs, while *ODC*-derived PAs are required for subsequent growth and development of citrus fruits.

Spermidine content decreased across the flowering stages (Figure 6) and it continued to drop throughout all the fruit set stages, regardless the cultivar, year, and type of inflorescence [55,56], except at petal fall (growth stage 69 of BBCH-scale) when it slightly increased in the GA-induced fructification in the first-year experiment of [56]. The induction of spermidine content at the end of fruit set (growth stages 69 to 72 of BBCH-scale) could be due to the activity of SPDS enzyme, since the expression of *CsSPDS* displayed a transient decrease during the first week after anthesis, then recovered the initial level two weeks after anthesis and was significantly up-regulated three weeks after anthesis [56]. Likewise, tyramine demonstrated similar trends as spermidine throughout the fruit set period, where it suffered a transient drop until 21 days after anthesis (the growth stages 71-72 of BBCH-scale [56] (Figure 6). Interestingly, the reduction in spermidine and tyramine content might not be associated with the fruit set development, since both compounds were decreased in ovaries that had not accomplished fruit development at 21 days after anthesis [56]. Furthermore, spermidine content was higher in ‘Oronules’ Clementine (a cultivar with low fruit set) than in ‘Okitsu’ Satsuma mandarin (a cultivar with high fruit set) [55]. Likewise, spermidine content was significantly higher in low-flowering years than those for the high-flowering years [55]. Together, these findings suggest a negative correlation between fruit set and spermidine. Almost similar results were obtained from ‘Murcott’ mandarin, where putrescine biosynthetic enzymes (*CsADC* but not *CsODC*) and, to a lesser extent, putrescine and spermidine content roughly followed the patterns of fruit growth, with a higher content at the early stages, a minimum at mid-summer and intermediate values at full fruit development [71].

On the other hand, spermine content hardly changed and remained relatively constant during the first 14 days of fruit development after anthesis or until growth stage 69 of BBCH-scale, where it increased remarkedly and experienced a gradual increase [55,56]. However, the behavior of the spermine profile during the growth stage 71 and 72 of the BBCH-scale is controversial. For example, Trénor et al. (2010) showed that spermine content, from petal fall to the onset of physiological fruit drop, depending on the year that when the experiment was carried out since it decreased in the first-year experiment but slightly increased in the second-year experiment [56]. Likewise, Arias et al. (2005) reported that spermine content was decreased after the growth stage 69 of BBCH-scale regardless of the cultivar, year, or inflorescences type, except for ‘Oronules’ mandarin in the first-year experiment, where it slightly increased [55]. Studies on the annual cycle of endogenous PAs of two citrus varieties, ‘Washington’ navel orange (*C. sinensis*), and ‘Miyagawa’ Satsuma (*C. unshiu*), showed that change of the total PAs content in fruit paralleled the enlargement of fruit diameter and spermidine and spermine contents increased at later stages [151].

The induction of spermine levels could be due to the higher activity of SPMS. Interestingly, the transcript levels of *CsSPMS* was raised after the initiation of fruit set and maintained at this level until the growth stages 71-72 of BBCH-scale [55]. While the endogenous levels of spermine hardly changed during the first two weeks of fruit set, the content of synephrine and methyl-tyramine experienced a gradual increase [55]. However, the endogenous levels of synephrine and methyl-tyramine showed contrary results after petal fall through the growth stage 71-72 of the BBCH-scale. 

Furthermore, PAs levels were increased during fruit maturation and ripening of ‘Murcott’ mandarin [71]. These high endogenous PAs levels may be associated with the high growth rate [159] or active cell division [85]. We suggest that in the initial stage of fruit development, active cell division occurs, which possibly needs sufficient putrescine and spermidine contents. While, at the later stage of fruit development, cell division gives way to cell enlargement, in which PAs synthesis is enhanced to fulfill the nitrogen needs required for cell enlargement. Together these findings suggest that PAs might be used as a nitrogen source rather than a growth regulator of fruit set in citrus [55] since the fruit number positively correlated with the increased nitrogen supplementation if other factors are not limiting [55].

In addition to the well-known role of PAs in fruit set of citrus plants, they also could be involved in the fruitlet and fruit abscission [55,71,98], fruit quality [98], fruit creasing [160], alternate bearing [161], and postharvest storage life [162]. Briefly, the exogenous PAs supplementation decreases cell separation processes (abscission) of citrus fruit and fruitlet based on the polyamine compound, concentration, and time of application [55,71,98]. The foliar application of PAs during the flowering period significantly reduced the fruitlet abscission in ‘Murcott’ mandarin [71] and sweet orange [98] and decreased the fruit abscission percentage in ‘Oronules’ Clementine (*C. clementina*) and ‘Okitsu’ Satsuma (*C. unshiu*) mandarins [55].

Exogenous application of aqueous PA solutions significantly improved the fruit quality, represented by peel color, peel smoothness, total soluble solids (TSS), total and non-reducing sugars of ‘Blood Red’ sweet orange (*C. sinensis*). For example, spermidine application increased the TSS, total sugars, peel color, and peel smoothness score, while it reduced the fruit acidity [98]. These findings proved the effectiveness of spermidine for improving fruit quality of citrus plants. In addition, PAs are involved in creasing (albedo breakdown) of citrus fruits [160]. Creasing is a physiological disorder in the rind of sweet orange (C. sinensis) fruit and causes serious economic losses worldwide. Hussain and Singh (2015) found that the single-spray application of putrescine, at fruit set or golf ball stage, reduced creasing index percentage, improved the rind thickness, and increased the levels of endogenous PAs in the flavedo and albedo tissues of ‘Washington’ navel and ‘Lane Late’ fruit in a concentration-based manner. On the other hand, single-spray treatment of MGBG (PAs inhibitor) at the golf ball stage significantly increased the creasing index percentage in both cultivars [160].

Recently, Mirsoleimani and Shahsavar (2018) showed that the seasonal changes in the free PAs contents in the leaves and stems of ‘Kinnow’ mandarin (*C. reticulata*) could be involved in the alternate bearing habit for these plants. For instance, total PAs content in leaves was higher in “off” trees than in “on” trees throughout the flower bud formation period. Similarly, the endogenous levels of spermidine and spermine in the stems were significantly higher in “off” trees than in “on” ones most of the season, while putrescine content in the stems was significantly higher in “on” trees than in “off” ones [161]. Although these results did not prove the relationship between PAs changes in stems and leaves of citrus plants with processes of flower bud formation, these findings showed that alternate bearing habit could be controlled by variations in PAs content during the season.

## 9. Role of PAs in the Stomatal Closure and Gas-Exchange of Citrus Plants

Photosynthetic organisms, particularly green plants, require oxygen (O_2_) in order to carry on cellular respiration, while carbon dioxide (CO_2_) is required for photosynthesis. Unlike animals, plants do not have specialized organs for gas exchange. Therefore, plants exchange gases through their leaves via stomata. Although several studies showed that abiotic stresses increase the PA levels in the guard cells of stomata of several plant species [47,58,59,163,164,165], little is known about the physiological function of elevated PA levels in gas exchange of citrus plants. Generally, all-natural PAs strongly inhibited the opening and induced the closure of stomata but using different mechanisms.

For example, in *Vicia faba* plants, PAs regulate the voltage-dependent inward K^+^ channel in the plasma membrane of guard cells and modulate stomatal aperture [163]. While in *A. thaliana* plants, PAs regulates the stomatal closure through manipulation of the levels of NO as well as reactive oxygen species (ROS) in guard cells [165]. Similar results were obtained from ‘Red Tangerine’ (*C. reticulata*) plants, where the in vitro exogenous application of spermine (1 mM) resulted in higher endogenous PAs content which was associated with less wilted phenotype, lower water loss, and lower electrolyte leakage than the control plants [58]. Interestingly, the leaves of spermine-pretreated plants had less ROS and higher activities of peroxidase (POD) and superoxide dismutase (SOD) than the control [58]. Taken together, these findings suggest that PAs control the stomatal response of citrus plants via modulation of antioxidative capacity [58]. Another example of the association between PAs and ROS scavenging is that the overexpression of the abscisic acid-responsive element (*ABRE*)-binding factor (*ABF*) of *P. trifoliata* (*PtrABF*) significantly enhanced dehydration tolerance [59]. The *PtrABF* transgenic lines displayed smaller stomatal apertures and reduced stomatal density/index. Simultaneously, the transgenic plants had higher PAs content, which was associated with higher antioxidant enzyme activities and less ROS [59]. Taken together, these findings suggest that PAs might control the gas exchange and stomatal density via maintaining ROS homeostasis.

Addition of d-arginine (as PAs-biosynthesis inhibitor) to the saline HNS reduced the gas exchange-related parameters, including stomatal conductance, transpiration rate, intracellular CO_2_ concentration, and total putrescine content, but increased total spermine content of the attached leaves of ‘Cleopatra’ mandarin seedlings [57]. On the other hand, supplementation of the saline medium with spermidine (0.5 mM) alleviated the antagonistic effects of d-arginine and improved the total putrescine, spermidine, and spermine contents of the leaves [57]. Likewise, the application of putrescine inhibitor, DFMO, inhibited the stomatal conductance (*g*s) and transpiration rates (*E*) of *C. tangerine* seedlings [39]. However, the application of exogenous putrescine to DFMO-treated plants partly or completely reversed the negative effects of DFMO [39].

**Figure 7:**
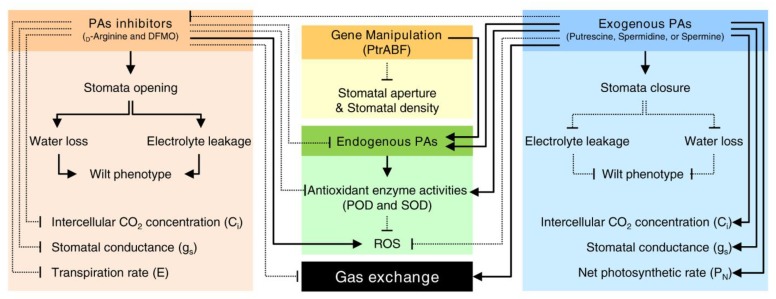
Hypothetical model of the effect of exogenous polyamines (PAs) and PAs biosynthesis inhibitors on gas exchange and its related parameters of citrus plants. Solid lines with arrows signify positive reaction, while round-dotted lines with bar-ends indicate negative interaction. Abbreviations: DFMO: α-difluoromethylornithine, *POD*: Peroxidase, *PtrABF:* Abscisic acid-responsive element (*ABRE*)-binding factor (*ABF*) of *Poncirus trifoliata*, ROS: Reactive oxygen species, and *SOD*: Superoxide dismutase. For more details, see the main text.

Recently it has been shown that exogenous application of PAs, particularly spermidine (0.5 mM), significantly increased the gas exchange parameters as presented by intercellular CO2 concentration (*C*i), stomatal conductance (*g*_s_), and net photosynthetic rate (*P*N) of Bakraii (*C. reticulata × C. limetta*) seedlings when subjected to salinity stress [47]. Additionally, boron deficiency in *C. sinensis* seedlings caused a significant reduction in some of gas exchange characteristics, but induction in other parameters. For example, boron-deficient lines exhibited lower leaf CO_2_ assimilation, leaf *g*s, leaf transpiration, and root respiration, but higher leaf *C*_i_ and dark respiration [62]. Interestingly, boron deficiency was associated with the accumulation of caffeoyl-PAs conjugates such as caffeoylputrescine and putative dicaffeoylspermidine in tobacco plants [166]. These findings indicate that PAs accumulation might be related to the gas exchange characteristics directly or indirectly (Figure 7); however, more investigations are required to understand the physiological role of elevated PAs levels in the modulation of gas exchange in citrus plants.

## 10. Role of PAs in the Photosynthesis and Chlorophyll Fluorescence of Citrus Plants

Although photosynthesis in higher plants is mainly controlled by three major factors including light irradiance, CO_2_ concentration, and temperature, it could be limited by a range of environmental factors such as leaf area, shading by other plants, water availability, and many other factors. Moreover, PAs are involved in regulating the light-harvesting and photosynthetic functions [61,167,168]. In citrus, many previous studies demonstrated the role of PAs in the photosynthesis, chlorophyll fluorescence, and their related parameters through the modifications of the endogenous levels of PAs [37,40,45,47,60,61,62].

For example, exogenously applied spermidine, by addition to the saline HNS or via weekly sprays, enhanced the accumulation of spermidine and spermine contents and improved chlorophyll content, net photosynthetic rate, and chlorophyll fluorescence yield (Fv/Fm; also known as maximum photochemical efficiency of PSII) of NaCl-stressed Troyer citrange plants [45]. Likewise, exogenously applied putrescine significantly increased chlorophyll a, total carotenoid, and total chlorophyll contents in trifoliate orange seedlings [37]. Moreover, the application of DFMO (a putrescine inhibitor) significantly decreased the total chlorophyll content and photosynthetic rates (*P*N) of *C. tangerine* seedlings, whereas the application of exogenous putrescine to DFMO-treated seedlings partly reversed the negative effects of DFMO [39]. Consistent with these findings, the application of different exogenous PAs (putrescine, spermidine, or spermine) improved the relative chlorophyll content in leaves of Canton lemon (*C. limonia*), which was associated with significantly increased in plant biomass. [40]. Taken together, these findings suggest that PAs might be positively influencing the photosynthetic functions of citrus plants. In fact, chloroplasts themselves could augment the endogenous PAs content, which can benefit the formation of chlorophyll *a* but not chlorophyll *b* [61]. However, the high dose of PAs may destroy the structure of chloroplasts based on light conditions [37]. 

Recently, the PAs-treated Iranian mandarin (*C. reticulata × C. limetta cv.* Bakraii) seedlings showed an increased net photosynthetic rate in salt-stressed plants, which was found to be associated with increased photosynthetic pigments content, as well as fluorescence parameters including minimal fluorescence yield of the dark-adapted state (F0), maximal fluorescence yield of the dark-adapted state (Fm), maximum photochemical efficiency of PSII (Fv/Fm), variable fluorescence (Fv), nonphotochemical quenching (NPQ), and photochemical quenching (qP) [47].

Under both saline and non-saline conditions, putrescine and paclobutrazol (a plant growth retardant and triazole fungicide) significantly enhanced the photosynthetic rates of salt-sensitive citrus rootstock Karna khatta (*C. karna*) seedlings [60]. In the same study, chlorophyll *a* and *b* were decreased significantly in NaCl-stressed plants, however, the application of putrescine, paclobutrazol, or a combination of both improved the chlorophyll *a*, chlorophyll *b*, total chlorophyll contents, and chlorophyll *a*/*b* ratio [60]. The positive effects of putrescine and paclobutrazol on chlorophyll could contribute directly toward the enhancement of photosynthesis in citrus. Paclobutrazol blocks some biosynthesis-limiting steps of the terpenoid/isoprenoid pathway which results in inhibiting the gibberellins biosynthesis but enhancing cytokinins and ABA accumulation [169,170]. The inhibition of gibberellin synthesis increases the availability of terpenoid pathway precursors, particularly geranylgeranyl diphosphate (GGPP). Increased GGPP could contribute directly toward the enhancement of chlorophyll content via phytyl-pp using geranylgeranyl reductase (*GGRS*), chlorophyll synthase (*CHL*), and some other enzymes [170]. Furthermore, it might play an indirect role in the enhancement of photosynthesis via the induction of carotenoids content through phytoene using phytoene synthase (*PSY*) [170]. Carotenoids play a photoprotective role in the photosynthetic process [171,172]. In addition to their role as accessory light-harvesting pigments, carotenoids protect chlorophylls from photo-damage through the absorption of light energy to be utilized in photosynthesis [171,172].

## 11. Conclusions Remarks and Future Prospects

The considerable increase in research studies about the roles of polyamines in citrus growth and development suggests that they do something interesting and important. In this review, we presented an overview of the role PAs in a wide range of physiological processes of citrus plants; however, their exact roles are not completely understood. Nevertheless, the knowledge gained thus far about the physiological roles of PAs in citrus builds a strong case for further studies toward understanding the metabolic and molecular regulation of PAs in citrus, and their emergence as a promising approach to practical applications in citriculture. Furthermore, several synergistic and antagonistic interactions exist between PAs and various phytohormones, particularly ABA and ethylene, during the regulation of cellular processes of citrus plants. Although significant progress has been made in understanding the crosstalk between PAs and phytohormones, the molecular mechanisms underlying PAs-phytohormone interactions require more investigation to understand the signaling transductions that regulate PA functions in citrus. 

In this review, we dissected the biosynthesis pathway of PAs in citrus plants, along with its associated biosynthetic genes. However, only two PAs biosynthetic genes have been cloned from citrus or its relatives, including *CsSAMDC* (GenBank Accession No. FJ496345) from ‘Newhall’ navel orange (*C. sinensis*) [173] and *PtADC* (GenBank accession No. HQ008237) from trifoliate orange (*P. trifoliata*) [74]. Further studies are required though for cloning, biochemical identification, and expression analysis of the genes involved in citrus PA biosynthesis. Traditional quantitative trait locus (QTL) mapping/cloning, and genome-wide association mapping are two of the best approaches that could be used for gene identification and modulation of PAs content by genetic engineering. The genetic manipulation of PAs biosynthetic genes to produce transgenic citrus plants with modulated PAs content may enhance the tolerance of citrus plants against stressful conditions. Moreover, isolation and analysis of PAs biosynthetic genes will significantly improve our understanding of the molecular mechanisms behind the role of PAs in stress tolerance. Transcriptomic, proteomic, and metabolomic approaches are very helpful tools to understand these molecular mechanisms. Additionally, the exogenous application of PAs can be an alternate option to enhance citrus tolerance, and other plant species in general, to both biotic and abiotic stresses.
